# Amlodipine versus angiotensin II receptor blocker; control of blood pressure evaluation trial in diabetics (ADVANCED-J)

**DOI:** 10.1186/1471-2261-6-39

**Published:** 2006-10-09

**Authors:** Ryuzo Kawamori, Hiroyuki Daida, Yasushi Tanaka, Katsumi Miyauchi, Akira Kitagawa, Dobun Hayashi, Junji Kishimoto, Shunya Ikeda, Yutaka Imai, Tsutomu Yamazaki

**Affiliations:** 1 Metabolism and Endocrinology, Department of Medicine, Juntendo University School of Medicine, 2-1-1 Hongo, Bunkyo-ku, Tokyo 113-8421, Japan; 2 Department of Cardiovascular Medicine, Juntendo University School of Medicine, 2-1-1 Hongo, Bunkyo-ku, Tokyo 113-8421, Japan; 3 Metabolism and Endocrinology, Department of Medicine, St. Marianna University School of Medicine, 2-16-1 Sugao, Miyamae-ku, Kawasaki-shi, Kanagawa 216-8511, Japan; 4 Department of Cardiovascular Medicine, Juntendo University School of Medicine, 2-1-1 Hongo, Bunkyo-ku, Tokyo 113-8421, Japan; 5 Clinical Trial Management, Graduate School, International University of Health and Welfare, 1-24-1 Minamiaoyama, Minato-ku, Tokyo 107-0062, Japan; 6 Department of Translational Research for Healthcare and Clinical Science, Graduate School of Medicine, The University of Tokyo, 7-3-1 Hongo, Bunkyo-ku, Tokyo 113-8655, Japan; 7 Digital Medicine Initiative, Department of Digital Organ, Kyushu University, 3-1-1 Maidashi, Higashi-ku, Fukuoka 812-8582, Japan; 8 Department of Pharmaceutical Sciences, International University of Health and Welfare, 2600-1 Kitakanemaru, Otawara-shi, Tochigi 324-8501, Japan; 9 Department of Clinical Pharmacology and Therapeutics, Tohoku University Graduate School of Pharmaceutical Sciences and Medicine, 6-3 Aoba, Aramaki, Aoba-ku, Sendai 980-8578, Japan; 10 Department of Clinical Bioinformatics, Graduate School of Medicine, Faculty of Medicine, The University of Tokyo, 7-3-1 Hongo, Bunkyo-ku, Tokyo 113-8655, Japan

## Abstract

**Background:**

The coexistence of type 2 diabetes mellitus and hypertension increases the risk of cardiovascular diseases. The U.K. Prospective Diabetes Study has shown that blood pressure control as well as blood glucose control is efficient for prevention of complications in hypertensive patients with diabetes mellitus. However, some reports have shown that it is difficult to control the blood pressure and the concomitant use of a plurality of drugs is needed in hypertensive patients with diabetes mellitus. In recent years renin-angiotensin system depressants are increasingly used for the blood pressure control in diabetic patients. Particularly in Japan, angiotensin II (A II) antagonists are increasingly used. However, there is no definite evidence of the point of which is efficient for the control, the increase in dose of A II antagonist or the concomitant use of another drug, in hypertensive patients whose blood pressure levels are inadequately controlled with A II antagonist.

**Methods/Design:**

Hypertensive patients of age 20 years or over with type 2 diabetes mellitus who have been treated by the single use of AII antagonist at usual doses for at least 8 weeks or patients who have been treated by the concomitant use of AII antagonist and an antihypertensive drug other than calcium channel blockers and ACE inhibitors at usual doses for at least 8 weeks are included.

**Discussion:**

We designed a multi-center, prospective, randomized, open label, blinded-endpoint trial, *ADVANCED-J*, to compare the increases in dose of A II antagonist and the concomitant use of a Ca-channel blocker (amlodipine) and A II antagonist in hypertensive patients with diabetes mellitus, whose blood pressure levels were inadequately controlled with A II antagonist. This study is different from the usual previous studies in that home blood pressures are assessed as indicators of evaluation of blood pressure. The *ADVANCED-J *study may have much influence on selection of antihypertensive drugs for treatment in hypertensive patients with diabetes mellitus. It is expected to give an important hint for considering the validity of selection of antihypertensive drugs from the aspects not only of the antihypertensive effect but medical cost-effectiveness.

## Background

It has been revealed by many epidemiological studies including the Framingham study that diabetes mellitus (DM) and hypertension (HT) are respectively risk factors of cardiovascular diseases and that the coexistence of DM with HT considerably increases the risk of cardiovascular diseases [1-4]. The results of the U.K. Prospective Diabetes Study (UKPDS) suggest that blood pressure control, rather than blood glucose control, is efficient for prevention of macrovascular complications of those of DM, which include stroke and myocardial infarction[5]. The results of the Hypertension Optimal Treatment (HOT)-study on the correlation between optimum target blood pressure levels and the occurrence of cardiovascular events also suggest that it is useful for HT patients with DM to set the target levels lower than those for general HT patients[6]. Aggressive antihypertensive therapy needs to be carried out.

On the basis of these knowledge, observations, and findings, optimum target blood pressure levels for HT patients with DM (DM+HT patients) are set at 130/80 mm Hg lower than those for general HT patients in various guidelines [7-10]. While optimum target blood pressure levels for DM+HT patients are set at lower levels, it is known that it is difficult to control blood pressure in these patients. The results of many large-scale clinical studies have shown that the combined use of a plurality of antihypertensive drugs is actually needed to blood pressure control.

The types of antihypertensive drugs that are recommended to the treatment of DM+HT patients vary with guidelines, but in many cases renin-angiotensin (RA) system depressants and calcium channel blockers (Ca blockers) are recommended, taking into consideration the influence on glucose metabolism.

Angiotensin II (A II) is a peptide hormone closely involved with the Na excretion control via the RA system. A II is widely recognized from the action mechanism to influence the onset and exacerbation of HT. ACE inhibitors suppressing A II production and A II receptor antagonists (A II antagonists) have been developed as antihypertensive drugs suppressing the RA system, and used all over the world [5,11,12]. It has also been shown that A II has an adverse influence on carbohydrate metabolism. These RA system depressants may also be expected to improve glucose tolerance in DM patients, and the frequency of the drugs used is being increased [13-18].

On the other hand, Ca antagonists exert the antihypertensive action to wide-ranging patients, and are commonly used as antihypertensive drugs through the mechanism of inhibiting calcium entry, which triggers constriction in vascular smooth muscle cells. Ca antagonists are recommended as a therapeutic medicine for DM+HT patients, because they have no adverse influence on lipid metabolism or glucose metabolism [19-28].

In recent years, the use of A II antagonists as antihypertensive drugs for DM+HT patients is being increased in Japan. The increase in dose of antihypertensive drug, the combined use of antihypertensive drugs with different mechanisms, and so on, are considered as methods to respond to the inadequate control of blood pressure in HT patients. However, there is no distinct evidence of the measure that will make better control of blood pressure to become a reality in DM+HT patients, whose blood pressure is inadequately controlled with A II antagonist.

In hypertensive patients whose blood pressure levels were inadequately controlled by single therapy with an A II antagonist, we designed the ADVANCED-J study to compare different methods for antihypertensive therapy, i.e., the increase in dose of A II antagonist and the combined use of a Ca blocker amlodipine, which is most frequently used in Japan, in terms of the usefulness.

## Methods/Design

### Study population

Subjects are recruited in outpatient offices of university hospitals or general hospitals from the entire apectrum of patients with coexistent hypertension and type 2 diabetes mellitus.

#### Inclusion criteria

The patients, who are judged as meeting *all *of the following conditions by investigators, are included in the study.

1) Patients with type 2 diabetes mellitus

2) Patients who have been treated by the single use of AII antagonist (Nu-lotan Tablet, i.e., a losartan potassium preparation, is used in the patients who start receiving treatment from the observation period) at usual doses for at least 8 weeks (including the observation period of 2 weeks or more for the study) or patients who have been treated by the concomitant use of AII antagonist and an antihypertensive drug other than calcium channel blockers and ACE inhibitors at usual doses for at least 8 weeks (including the observation period of 2 weeks or more for the study).

3) Patients who show > 135 mm Hg systolic or > 85 mm Hg diastolic of the ambulatory blood pressure levels measured (in the sitting position) at the time of start of the observation period, and show > 130 mm Hg systolic or > 80 mm Hg diastolic of the mean (home blood pressure level measured after getting up before the start of study) of the home blood pressure levels after getting up for the last 5 days during the last 2-week observation period.

4) Patients whose consent is obtained at age 20 years or over.

5) Patients whose consent is obtained from themselves in written form.

With regard to 2) and 3), however, the case, which shows > 180 mm Hg systolic or > 110 mm Hg diastolic of the mean of the home blood pressure levels measured after getting up for the last 5 days during the first 1-week observation period, is included in the study.

#### Exclusion criteria

This study excludes the patients who are judged as being conformed to *one *of the following conditions by investigators.

1) Patients with secondary hypertension.

2) Patients who show > 180 mm Hg systolic or > 110 mm Hg diastolic of the ambulatory blood pressure levels measured (in the sitting position) at the time of start of the observation period.

3) Patients with severe hepatic dysfunction.

4) Patients with severe renal dysfunction.

5) Patients with a past history of hypersensitiveness to study drugs.

6) Pregnant, lactating, and probably pregnant patients, and patients who want to become pregnant during the study period.

7) Patients who have attended other trials within 3 months before the start of the observation period or who attend other trials simultaneously with the present study.

8) Other patients judged as being inappropriate for the subjects of the study by investigators.

### Allocation procedure

Investigators confirm that the subjects are consistent with the inclusion criteria and are not contrary to the exclusion criteria and input necessary matters on the Web before the start of the study (before the start of the period when the dose is increased or other concomitant drug is used). The results of the dynamic allocation to two groups according to the eligibility of the relevant patient to the study and background factors (Increased AII antagonist dose group and combined amlodipine group) are indicated on the Web by E-mail.

The urinary albumin levels during the observation period (< 300 mg/g Cre, > 300 mg/g Cre) and systolic blood pressure levels (< 135 mm Hg, 135 mm Hg ≦) of the home blood pressure levels after getting up before the start of the study are taken into consideration on allocation of the patients (into the increased AII antagonist dose group and the combined amlodipine group).

### Study drugs

The following commercially-available drugs are used as study drugs in the study (See Table 1,2):

**Table 1:** List of AII receptor antagonists

Generic name	Study drug name (Brand name)	Active ingredient content	Manufacturers/distributors
candesartan cilexetil	Blopress^® ^tablets 2	containing 2 mg of candesartan cilexetil	Takeda Pharmaceutical Co., Lid.
	Blopress^® ^tablets 4	containing 4 mg of candesartan cilexetil	
	Blopress^® ^tablets 8	containing 8 mg of candesartan cilexetil	
	Blopress^® ^tablets 12	containing 12 mg of candesartan cilexetil	

losartan potassium	Nu-lotan^® ^tablets 25	containing 25 mg of losartan potassium	Banyu Pharmaceutical Co., Ltd.
	Nu-lotan^® ^tablets 50	containing 50 mg of losartan potassium	

telmisartan	Micardis^® ^capsules 20 mg	containing 20 mg of telmisartan	Nippon Boehringer Ingelheim Co., Ltd./Yamanouchi Pharmaceutical Co., Ltd. ^1)^
	Micardis^® ^capsules 40 mg	containing 40 mg of telmisartan	

valsartan	Diovan^® ^tablets 20 mg	containing 20 mg of valsartan	Nihon Chiba-Geigy K.K./Novartis Pharma K.K.
	Diovan^® ^tablets 40 mg	containing 40 mg of valsartan	
	Diovan^® ^tablets 80 mg	containing 80 mg of valsartan	

olmesartan medoxomil	Olmetec^® ^tablets 10 mg	containing 10 mg of olmesartan medoxomil	Sankyo Co., Ltd.
	Olmetec^® ^tablets 20 mg	containing 20 mg of olmesartan medoxomil	

1) After April 1, 2005, Astellas Pharma Inc.

**Table 2:** List of calcium channel blocker

Generic name	Study drug name (Brand name)	Active ingredient content	Manufacturers/distributors
amlodipine besilate	Amlodin^® ^tablets 2.5	containing 2.5 mg of amlodipine	Sumitomo Pharmaceuticals Co., Ltd.
	Amlodin^® ^tablets 5	containing 5 mg of amlodipine	
	Norvasc^® ^tablets 2.5 mg	containing 2.5 mg of amlodipine	Pfizer Japan Inc.
	Norvasc^® ^tablets 5 mg	containing 5 mg of amlodipine	

#### Dosage regimen of study drugs and administration period

##### (1) Dosage regimen

With regard to the time of oral administration (direction for use), the study drug is taken after breakfast or after getting up once a day.

The dose of AII antagonist in the increased AII antagonist dose group is the maximum dose approved. However, in the cases in which the antihypertensive effect is excessive (< 110 mm Hg systolic or < 65 mm Hg diastolic of the home blood pressure after getting up), and in which the dose is required to be reduced, the dose of AII antagonist may be reduced.

The dose of AII antagonist in the combined amlodipine group is the same as that (usual dose) during the observation period, and the dose of amlodipine is 5 mg/day. However, in the cases in which the antihypertensive effect is excessive (< 110 mm Hg systolic or < 65 mm Hg diastolic of the home blood pressure* after getting up) and in which the dose is required to be reduced, the dose of amlodipine may be reduced (See Table 3).

**Table 3:** Dosage regimens of study drugs for two groups

Study drug	Dosage and administration (oral)	Usual daily dose	
		
		Increased AII antagonist dose group	Combined amlodipine group
Blopress^® ^tablets	o.d.	12 mg	8 mg
Nu-lotan^® ^tablets	o.d.	100 mg	50 mg
Micardis^® ^capsules	o.d.	80 mg	40 mg
Diovan^® ^tablets	o.d.	160 mg	80 mg
Olmetec^® ^tablets	o.d.	40 mg	20 mg
Amlodin^® ^tablets, or Norvasc^® ^tablets	o.d.	n/a	5 mg

1) The approved maximum daily dose

2) The upper limit of the usual daily doses approved for AII antagonists

* Note: The mean of the blood pressure levels measured for the last 5 days during the 2-week period throughout the study period.

##### (2) Administration period

Three years after the start of study.

### Target blood pressure levels

The home blood pressure levels after getting up are < 125 mm Hg systolic and < 80 mm Hg diastolic.

* **Note: **The mean of the levels measured for the last 5 days during the 2-week period (during the 14-day period between 14 days before the day of ambulatory examination and the previous day of the ambulatory examination day) before the ambulatory examination day (the reference day of the months without ambulatory examination).

### Other concomitant drug and combined therapy

(1) The following drugs may be concomitantly used with AII antagonist under individual conditions

1) Antihypertensive drugs used concomitantly with AII antagonist before the start of the observation period (The drugs other than calcium channel blockers and ACE inhibitors)

2) Antihypertensive drugs other than the study drug from 8 weeks onward after the start of the study.

3) Drugs used for the treatment of complications

(2) The drugs prohibited to be concomitantly used

Antihypertensive drugs other than the study drug for 8 weeks after the start of the study.

(3) Combined therapy

Combined therapy including standard diet therapy, therapeutic exercise, prohibition of smoking, etc. is not restricted.

### Steps to increase doses of the study drug and antihypertensive drugs other than the study drug and to use concomitantly an antihypertensive drug other than the study drug

**Figure 1:**
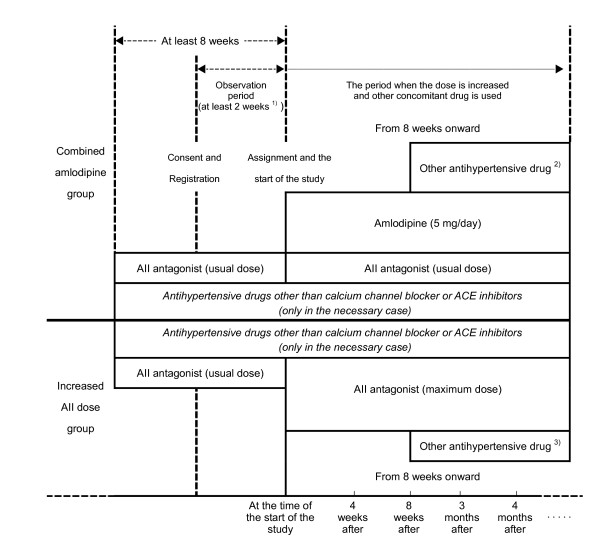
Steps to increase doses of study drug and antihypertensivedrugs other than the study drug and to use other concomitant drug. 1) In the case in which the systolic blood pressure (mean level of the levels measured for last 5 days) measured at home after getting up for a week after the start of observation period is > 180 mm Hg or the diastolic blood pressure level is > 110 mm Hg, the case may be assigned to the treated group. 2, 3) Only when the blood pressure level does not reach the target systolic and diastolic blood pressure measured at home after getting up are < 125 mm Hg and < 80 mm Hg, respectively). 2) The combined use of renin-angiotensin inhibitors (ACE inhibitors, AII antagonist) is prohibited. 3) The combined use of calcium channel blocker is prohibited.

A chart of steps to increase doses of the study drug and antihypertensive drugs other than the study drug and to use concomitantly an antihypertensive drug other than the study drug is shown below (See Figure 1).

### Study period

Between the time of obtainment of the patients' consent and the end of the (3-year) period of the increase in dose or the concomitant use (However, the 2-year period from one year onward after the start of the study is the follow-up study period).

### Outcome measures

#### (1) Outcome measures for efficacy

##### Main outcome measures

• Changes in the home blood pressures after getting up (home blood pressure levels)

• The rate of home blood pressure levels accomplishing the target levels (< 125 mm Hg systolic and < 80 mm Hg diastolic)

##### Secondary outcome measures

• Changes in the blood pressure levels measured on an outpatient basis (ambulatory blood pressure level)

• The rate of the ambulatory blood pressure levels accomplishing the reference levels (< 130 mm Hg systolic and < 80 mm Hg diastolic)

• Changes in the home blood pressures before going to bed

• Changes in IMT of the cervical artery

• Changes in PWV

• Changes in echocardiographic findings

• Changes in urinary albumin level

• Changes in BNP

• Changes in hs-CRP

• Medical cost-effectiveness

#### (2) Outcome measures for safety

• Adverse events, adverse drug reactions

• Clinical laboratory data

### Statistical analysis

#### (1) Transaction of cases

The Clinical Protocols Committee and members of the Clinical Protocols Preparation Subcommittee decide the transaction of the cases and to fix the data collected for 12 months and 36 months after the start of the study.

#### (2) Population to be analyzed

1) Population to be analyzed for efficacy

• The major population to be analyzed for efficacy includes those defined according to the principle of intention-to-treat principle.

2) Population to be analyzed for safety

• The population to be analyzed for safety include the population in which the dose of the AII antagonist is increased or the additional concomitant use of amlodipine is conducted after the start of the study.

#### (3) Statistical analysis design

Main outcome measures and analytical methods:

1) Main outcome measures

(a) Changes in the home blood pressures after getting up (home blood pressure level)

(b) The rate of home blood pressure levels accomplishing the target levels

2) Analytical methods

With regard to (a), the subjects include "the population to be analyzed for efficacy", and summarized statistics about difference in blood pressure (the extent of antihypertensive effect) at each instant of survey are calculated from blood pressure at each instant of survey and from blood pressure levels during the observation period. Analysis of variance is applied for the inter-group comparison. In each group the blood pressure levels during the observation period are compared with the blood pressure levels at the instant of survey for reference. The extent of antihypertensive effect is also compared between the groups at each instant of survey. With regard to (b), the subjects include "the population to be analyzed for efficacy", and the rates of the blood pressure levels measured 8 and 12 months after the start of the study, which accomplish the target blood pressure levels, are calculated and the frequencies with which the rates are obtained are compared by χ^2 ^test.

Secondary outcome measures and analytical methods:

1) Secondary outcome measures

(a) Changes in ambulatory blood pressure levels

(b) The rate of ambulatory blood pressure levels accomplishing the reference levels

(c) Changes in home blood pressure levels measured before going to bed

(d) Changes in carotid IMT

(e) Changes in PWV

(f) Changes in echocardiographic findings

(g) Changes in urinary albumin level

(h) Changes in BNP

(i) Changes in hs-CRP

(j) Medical cost-effectiveness

2) Analytical methods

With regard to items (a) and (c), the analysis includes "the population to be analyzed

for efficacy", and conforms to " (3) 1) (a) Changes in blood pressure levels measured

at home after getting up". With regard to item (b), the analysis includes "the

population to be analyzed for efficacy", and conforms to "(3) 1) (b) The rate of home

blood pressure levels accomplishing the target levels". With regard to items (d)

through (i), the analysis includes "the population to be analyzed for efficacy", and the

blood pressure levels during the observation period are compared with those at each

time point of survey in each group. The difference between the blood pressure level

during the observation period and that at each instant of survey is compared

between the groups. With regard to item (j), the analysis includes "the population to

be analyzed for efficacy", and the drugs used, the contents of events, and medical

fees required are compiled according to groups for comparison.

### Analysis period

The statistical analysis follows the fixation of the data collected for 12 months after the start of the study and of the data collected (for 36 months after the start of the study) during the follow-up period.

### Target number of the patients

The target number of the patients is 300 patients consisting of 150 in the increased AII antagonist dose group and 150 in the combined amlodipine group.

## Discussion and conclusion

### Significance of the ADVANCED-J study

Selection of methods for antihypertensive therapy has been a matter of controversy, but the results of meta-analysis of the Blood Pressure Lowering Treatment Trialists' Collaboration (BPLTTC), in which the results of large-scale clinical studies in recent years were comprehensively interpreted, have suggested that the way to decrease the blood pressure, rather than the types of antihypertensive drugs, is important for prevention not only of stroke but of coronary arterial diseases such as myocardial infarction. When the efficacy of the initiated antihypertensive drug is inadequate, physicians adopt either the increase in dose of the antihypertensive drug or the combined use of another antihypertensive drug with the initiated drug as alternative choices. However, there are hardly any studies on comparison of these alternatives in terms of the antihypertensive effect or the influence on cardiovascular events.

Some reports have shown that the combined use of antihypertensive drugs is needed to particularly HT patients with DM or nephropathy. Of these drugs, A II antagonists have been reported to have favorable influence on the renal function. In recent years the use of A II antagonists is increasingly used as the first-line medicine for HT patients with DM or nephropathy. On the other hand, it is also true that there are many patients who receive treatment by the combined use of a Ca blocker with the antagonist in many studies of evaluation of the efficacy of these drugs. The results of the meta-analysis in recent years have suggested that adequate control of blood pressure levels is also necessary for adequate exertion of the usefulness of RA system depressants in hypertensive patients.

While it has been reported that there is correlation between the antihypertensive action and the dose of A II antagonists, some reports have shown that the increase in the antihypertensive effect is limited even by the increase in dose to more than the usual dose. Since an adverse effect of ACE inhibitors, a dry cough, is frequently observed in Japanese people, the shift to A.R.B. rapidly proceeded. However, NHI drug price of A.R.B. is high. With regard to the use at dose more than the usual dose, its efficacy needs to be reviewed from a viewpoint of medical cost-effectiveness. Based on the background, the ADVANCED-J study was designed to compare the increase in dose of A II antagonists and the combined use of amlodipine with an antagonist in terms of the antihypertensive effect in hypertensive patients whose blood pressure levels were inadequately controlled by single therapy with an A II antagonist.

### Assessment of the home blood pressure

The primary outcome measure of the ADVANCED-J study included the home blood pressure measured in the early morning. The prevalence of home blood pressure monitor in recent years has allowed comprehension of details of the blood pressure condition. As a result, the presence of so-called masked hypertension, which shows the inadequately controlled home blood pressure in spite of the controlled ambulatory blood pressure, has increasingly been revealed.

It has been reported that the risk of cardiovascular events in masked hypertension patients is as high as that in persistent hypertension patients. The importance of 24-hour blood pressure control involving home blood pressure is increasingly recognized. In fact, it is not realistic on a point of principle or from the aspect of medical cost-effectiveness to conduct the ambulatory blood pressure monitoring (ABPM) in all hypertensive patients. On the other hand, at least 30,000,000 home blood pressure monitors have been put on the market up to date in Japan, and it can be said from the global aspect that they have become more widespread in Japan. The blood pressure levels measured with home blood pressure monitors, particularly those measured at the time of rising in the morning, have been reported to reflect changes in blood pressure during the course from the night to the early morning. The correlation between blood pressure levels at home in the morning and the onset of cardiovascular events has also been reported. It is considered important for suppression of cardiovascular events in HT patients with DM to control the home blood pressures measured in the morning.

There are many DM patients who show the non-dipper type of changes in blood pressure, i.e., the type which shows an only slight spontaneous decrease in blood pressure during the night (because of poor control of the autonomic nervous system and of renal hypofunction), and the home blood pressures measured in the morning are predicted to be high in them. It is therefore considered important for prevention of the onset of cardiovascular events to control the home blood pressure measured in the morning even in HT patients with DM.

However, there have been only few or no comparative studies on drugs, which have focused on home blood pressures measured in the morning in HT patients with DM. There have been no studies on the point of which is more efficient for controlling of home blood pressures measured in the early morning, the increase in dose of A II antagonists or the additional combined use of a Ca blocker. Based on these circumstances, the home blood pressures in the morning were adopted to evaluation of blood pressure control in this study.

The compliance of recording by patients and bias due to selection of levels at the time of data recording have influence on reliability of the measurement of blood pressure levels at home. For this reason, the i-monitoring system was adopted to the ADVANCED-J study, and all the blood pressure levels measured were semiautomatically accumulated in the Data Center. All the data from the measurements and the summary data were supplied to physicians via web when necessary.

### Assessment of arteriosclerosis

It has been believed that arteriosclerosis is more likely to progress in DM patients. According to Kawamori et al., it is clear that intimal-medial thickening (IMT), i.e., an indicator of systemic arteriosclerosis, has been increased even in the pre-stage of DM (IFG, IGT). As methods of assessing arteriosclerosis in the coronary artery, various techniques, such as quantitative coronary arteriography, intravascular ultrasonography, multi-slice CT, MRI, etc., have been developed. It is impossible to widely use these techniques because of some problems with invasion to patients, exposure to radiation and magnetism, etc. and of procedural complexity of measurement. The assessment of IMT by cervical arterial echography or the evaluation of baPWV by measurements of pulse waves at the wrist and ankle yields no invasion to patients. These techniques are methods of assessing arteriosclerosis, which rarely allow physical load to patients. Even for general clinics, these techniques are the less invasive methods for assessment, which are simple to maneuver. IMT and baPWV were applied as indicators in this study, and the influence of antihypertensive therapy on progression of arteriosclerosis was also evaluated.

## Conclusion

The ADVANCED-J study is a prospective clinical study that is designed to compare the increase in dose of A II antagonist and the combined use of a Ca blocker, amlodipine, in terms of the efficacy for blood pressure control in DM+HT patients whose blood pressure levels are inadequately controlled by the single treatment with an A II antagonist. This study is different from the usual previous studies in that home blood pressures are assessed as indicators of evaluation of blood pressure.

The ADVANCED-J study may have much influence on selection of antihypertensive drugs for treatment in HT patients with DM. It is expected to give an important hint for considering the validity of selection of antihypertensive drugs from the aspects not only of the antihypertensive effect but medical cost-effectiveness.

## Competing interests

The author(s) declare that they have no competing interests.

## Authors' contributions

RK and HD functioned as the general control physicians. JK drafted the statistical analysis plan. YI contributed to set out standard blood pressure levels. TY took the initiative in designing study protocol. YT, KM, AK, DH and SI also participated cooperatively to design study protocol. All authors read and approved the final manuscript.

## Appendix

### Authors

Ryuzo Kawamori(Metabolism and Endocrinology, Department of Medicine, Juntendo University School of Medicine); Hiroyuki Daida(Department of Cardiovascular Medicine, Juntendo University School of Medicine); Yasushi Tanaka(Metabolism and Endocrinology, Department of Medicine, St. Marianna University School of Medicine); Katsumi Miyauchi(Department of Cardiovascular Medicine, Juntendo University School of Medicine); Akira Kitagawa(Clinical Trial Management, Graduate School, International University of Health and Welfare); Dobun Hayashi(Department of Translational Research for Healthcare and Clinical Science, Graduate School of Medicine, The University of Tokyo); Junji Kishimoto(Digital Medicine Initiative, Department of Digital Organ, Kyushu University); Shunya Ikeda(Department of Pharmaceutical Sciences, International University of Health and Welfare); Yutaka Imai(Department of Clinical Pharmacology and Therapeutics, Tohoku University Graduate School of Pharmaceutical Sciences and Medicine); Tsutomu Yamazaki(Department of Clinical Bioinformatics, Graduate School of Medicine, Faculty of Medicine, The University of Tokyo) for ADVANCED-J Study Group

### ADVANCED-J study group investigators

Juntendo University School of Medicine: *Control physician: *Ryuzo Kawamori(Metabolism and Endocrinology, Department of Medicine); Hiroyuki Daida(Department of Cardiovascular Medicine); *Responsible physician: *Hirotaka Watada(Metabolism and Endocrinology, Department of Medicine); Katsumi Miyauchi(Department of Cardiovascular Medicine); *Participating physician: *Takahisa Hirose(Metabolism and Endocrinology, Department of Medicine); Hiromasa Suzuki(Department of Cardiovascular Medicine); Takeshi Kurata(Department of Cardiovascular Medicine); Tetsuro Miyazaki(Department of Cardiovascular Medicine); *IMT in charge: *Emi Miyazawa(Metabolism and Endocrinology, Department of Medicine); Noriko Iijima(Metabolism and Endocrinology, Department of Medicine); *CRC: *Atsuko Shiratori; Takagi Hospital: *Responsible physician: *Yasukazu Sato; *Participating physician: *Chiyoko Endo; Kaoru Tanaka; Keiki Yoshida; Kimihiro Nakahara; Kyosuke Yamamoto; Masahito Sakai; Masao Ohashi; Noriaki Matsumoto; Noriko Fukushima; Rieko Sakamoto; Satomi Fujimatsu; Yasuhiro Ono; *CRC: *Junko Matsuda; Kumiko Sasaoka; Noriko Takemata; *Clerical work: *Eisuke Mizokami; Hidenori Seki; International Hospital of Health and Welfare: *Responsible physician: *Fumio Murayama; *Participating physician: *Kenichi Oya ; Masao Hiwatari; *CRC: *Yasuko Otsuki; *Clerical work: *Kazuya Murakami; Kanazawa Medical Clinic: *Participating physician: *Shunichi Tanaka; *CRC: *Keiko Kikuchi; IUHW Atami Hospital: *Responsible physician: *Motoo Tsushima; *Participating physician: *Eiko Ikoma; Hisaichiro Tsukiyama; Izumi Kobayashi; Jin Oshikawa; Keiichi Shimoda; Kozo Okada; Ryu Sasaki; Takashi Orii; Tetsuya Fujikawa; Tomohiko Shigemasa; Yoshihiko Yamada; *CRC: *Asei Kaji; Junko Yamanaka; Takahiro Suzuki; Yoshiko Harakawa; *Clerical work: *Yoshiyuki Sato; Juntendo University Shizuoka Hospital: *Responsible physician: *Masahiko Kawasumi; Satoshi Kojima; *Participating physician: *Akihisa Nishino; Fuki Ikeda; Hidenori Yoshii; Masataka Niwa; Satoru Suwa; *CRC: *Atsushi Kobayashi; Takuya Uematsu; Yoshiaki Matsui; Juntendo University Urayasu Hospital: *Responsible physician: *Tatsuji Kanoh; *Participating physician: *Jong Bock Choi; Kenji Watanabe; Shigeru Matsuda; Takayuki Yokoyama; *CRC: *Miki Kubota; Shigeko Oyanagi; Sanno Hospital: *Responsible physician: *Kazuhide Yamaoki; Ikuo Yokoyama; Noriko Tajima; Wataru Hayashida; *CRC: *Chiyako Minamikawa; Sanno Medical Plaza: *Participating physician: *Atsurou Kishimoto; Tetsu Matsubara; Tomoko Yamazaki; *Clerical work: *Koichi Maeda; Kurata Clinic: *Responsible physician: *Takeshi Kurata; Nakakinen Clinic: *Responsible physician: *Takeshi Osonoi; *Participating physician: *Miyoko Saitou; Naoki Owada; Naoko Takayanagi; Shimada General Hospital: *Responsible physician: *Issei Shimada; *Clerical work: *Mika Wakaumi; Chiba Tokusyukai Hospital: *Responsible physician: *Michiko Abe; *Participating physician: *Miki Takagi; Takahisa Hirose; Koto Hospital: *Responsible physician: *Eiji Tamiya; IUHW Mita Hospital: *Responsible physician: *Kunio Ohyama; *Participating physician: *Atsuhisa Sato; Hideki Koh; Katsumasa Yui; Kunio Nakano; Yoshitaka Akiyama; *CRC: *Tomoko Shigeoka; National Hospital Organization Ureshino Medical Center: *Responsible physician: *Shiro Hata; Fukuoka Chuo Hospital: *Responsible physician: *Masao Ohashi; International Goodwill Hospital: *Responsible physician : *Akio Kanazawa; *Participating physician: *Yasuhiro Amagasaki; Ochanomizuhijiribashi Clinic: *Responsible physician: *Koshiro Monzen; The University of Tokyo Hospital: *Responsible physician: *Tsutomu Yamazaki; *Participating physician: *Dobun Hayashi; Koshiro Monzen; Ryozo Nagai; Takahide Kohro; Yasushi Imai; Arisaka Clinic: *Responsible physician: *Tomoyuki Arisaka; Hatori Medical Clinic: *Responsible physician: *Hiroshi Hatori; Hattori Clinic: *Responsible physician: *Akira Hattori; Hayashi Medical Clinic: *Responsible physician: *Yoshitaka Hayashi; Iwase Clinic of Cardiology and internal medicine:*Responsible physician: *Takashi Iwase; Junseikai Hospital: *Participating physician: *Kohji Komiya; Tomoaki Yoshihara; Yoshifumi Tamura; Kitanarashino Hanawa Hospital: *Responsible physician: *Mikio Tanaka; Matsumoto Clinic: *Responsible physician: Y*oshihisa Matsumoto; Mizuma Kouhoukai Hospital: *Responsible physician: *Masayasu Higashijima; *CRC: *Noriko Takemata; *Clerical work: *Hideki Nogami; Masayuki Matsufuji; Clinical Trial Management, Graduate School, International University of Health and Welfare: *CRC: *Ayako Nakahara; Kaoru Hatanaka; Mika Nozawa; Taka Ikarashi; *Clerical work: *Hideka Ishii; Kazuo Takeshita; Mari Kanno; Masami Yoshida

### ADVANCED-J participating facilities

Metabolism and Endocrinology, Department of Medicine, Juntendo University School of Medicine; Department of Cardiovascular Medicine Juntendo University School of Medicine; Juntendo University Urayasu Hospital; Juntendo University Shizuoka Hospital; Chiba Tokusyukai Hospital; Junseikai Hospital; Sanno Hospital; Sanno Medical Plaza; Takagi Hospital; Mizuma Kouhoukai Hospital; IUHW Atami Hospital; International Hospital of Health and Welfare; Iwase Clinic of Cardiology and Internal Medicine; Ochanomizu Hijiribashi Clinic; The University of Tokyo Hospital; Nakakinen Clinic; Shimada General Hospital; Koto Hospital; Hatori Medical Clinic; Kurata Clinic; Matsumoto Clinic; Kitanarashino Hanawa Hospital; Hayashi Medical Clinic; Arisaka Clinic; International Goodwill Hospital; IUHW Mita Hospital; Kanazawa Medical Clinic; Hattori Clinic; Fukuoka Chuo Hospital; National Hospital Organization Ureshino Medical Center

## Pre-publication history

The pre-publication history for this paper can be accessed here:


